# Natural Formulations Provide Antioxidant Complement to Hyaluronic Acid-Based Topical Applications Used in Wound Healing

**DOI:** 10.3390/polym12081847

**Published:** 2020-08-18

**Authors:** Pooyan Makvandi, Caterina Caccavale, Francesca Della Sala, Stefania Zeppetelli, Rosanna Veneziano, Assunta Borzacchiello

**Affiliations:** 1 Institute for Polymers, Composites and Biomaterials, National Research Council, IPCB-CNR, 80100 Naples, Italy; pooyan.makvandi@ipcb.cnr.it (P.M.); fr.dellasala@gmail.com (F.D.S.); stefania.zeppetelli@cnr.it (S.Z.); 2 Department of Chemical, Materials and Production Engineering, University of Naples Federico II, 80100 Naples, Italy; 3 University of Campania “Luigi Vanvitelli”, 81100 Caserta, Italy; caterina.caccavale93@gmail.com (C.C.); rosanna.veneziano@unicampania.it (R.V.)

**Keywords:** antioxidant properties, wound healing, rheology, hyaluronic acid

## Abstract

Hyaluronic acid (HA) promotes wound healing, and, accordingly, formulations based on HA have been widely used in regenerative medicine. In addition, naturally derived compounds, e.g., plant-based extracts and vitamin E, have exhibited antioxidant activity. In this study, a formulation containing hyaluronic acid, vitamin E, raspberry extract, and green tea was developed for potential topical applications, targeting wound healing. Rheological analysis was performed along with antioxidant and biological studies. The rheological characterization showed that the HA-based formulation is a thixotropic platform and possesses higher mechanical properties than the control formulation. To evaluate the wound healing potential of the formulation, an in vitro “wound healing” assay was carried out using human derived fibroblasts (HDF) with a cell-free gap on the tissue culture dish. The formulation showed better wound healing ability than the control formulation.

## 1. Introduction

Wound dressings can promote the healing process and reduce the healing time [1,2]. Hyaluronic acid (HA) is naturally occurring polysaccharide widely employed in regenerative medicine and in particular for topical and intradermal applications. HA is a biodegradable, biocompatible, non-toxic, non-immunogenic glycosaminoglycan, and it is present in mammalian connective tissues such as the dermis, synovial fluids, vitreous body, and nucleus pulposus where it fulfills important biophysical and biological functions. Hyaluronic acid promotes wound healing, and, accordingly, formulations containing HA have been applied in regenerative medicine. Natural polysaccharides such as HA have also been employed as drug carriers for biomedical applications along with many pharmaceutical formulations to enhance the biocompatibility of the final bioproduct [3,4,5]. It was shown that inhibiting oxidative stress in wounds can enhance the wound healing process. Therefore, antioxidant agents exhibit encouraging results in the wound healing process [6]. Radicals such as reactive oxygen species (ROS) play an important role in healing processes and in carcinogenesis. When the concentration of ROS goes beyond a certain threshold, cells and their compartments are damaged and, finally, they will die [7,8]. To overcome or, at least, diminish ROS production, which occurs during skin aging and cancer, attaining antioxidants from several sources, e.g., food supplements and skincare products, is taken into account increasingly [9,10]. Naturally derived antioxidants and vitamin E are commonly utilized in skincare products [11]. Among them, green tea and raspberry have shown strong antioxidant activity toward free radicals with possible implications in alleviating oxidative stress [12]. Additionally, vitamin E, because of its antioxidant activity, is frequently employed in drug delivery and wound healing applications [13].

HA can be employed to enhance skin regeneration for wound healing applications along with a carrier to locally deliver active biomolecules, such as antioxidants. Altogether, topical formulations possessing antioxidant properties and wound healing capability are great of interest to assist the healing of wounds such as abrasions [14,15,16,17]. The objective of this study was to create an enriched HA-based formulation with antioxidant activity containing vitamin E and natural compounds, such as raspberry and green tea, as a formulation to promote the healing process in topical applications. Since mechanical behavior in terms of rheological characterization can assist the tailoring and validation of the formulation for its biomedical and pharmaceutical functions, the platform rheological behavior—e.g., the thixotropy, viscosity, and strain-and-stress relationship—was evaluated. To further evaluate the formulation efficacy, anti-radical scavenging activity was assessed. Finally, biological properties, i.e., in vitro cytotoxicity and wound healing potential activity, were assessed.

## 2. Material and Methods

### 2.1. Materials

Hyaluronic acid (HA) with an average molecular weight (Mw) of 112 kg/mol was kindly provided by Altergon Italia. Vitamin E acetate (tocopherol acetate) was purchased from Sigma-Aldrich. The base formulation (control, named as Base) was purchased from Farmacia Vernile (Frosinone, Italy), with a composition of white Vaseline (25.5%), liquid semi-synthetic triglycerides (7.5%), cetylstearyl alcohol (6%), glycerylmonostearate (4%), polyethylene glycolstearate (7%), propylene glycol (10%), and purified water (40%); it is a base ingredient for making dermatological medication. High-quality green tea and raspberries were obtained from a local market. To obtain the raspberry juice, raspberries were processed with a blender (Philips HR2161/40, Amsterdam, The Netherlands) and the obtained juice was used.

### 2.2. Formulation Preparation

The HA-based natural formulation (HBN) was prepared by mixing green tea (2%) and vitamin E (0.75%) with the base formulation and mixing using a laboratory agitator (ATM, Falc instrument s.r.l., Treviglio, Italy) with a continuous regulation of speed (3000 rpm) for 30 min. HA (1% *w*/*w*) was first mixed with raspberry extract (1% *w*/*w*) and then added to green tea, vitamin E, and the base cream.

### 2.3. Physical Assessments and pH Determination

For the stability test, the formulation was left for up to 30 days at different temperatures, including in a refrigerator (4 °C), at room temperature, and in an oven (40 °C). During the storage, aspects of physical appearance such as phase separation, color, and fragrance were checked. The pH of each preparation was determined in diluted samples in bi-distilled water (1:10 *w*/*v*%), using Tornasol papers and a previously calibrated pH meter.

### 2.4. Rheological Analysis

#### 2.4.1. Oscillatory Rheometry

Small amplitude oscillatory shear tests were performed to evaluate the time-dependent response of the formulations and their linear viscoelastic properties, i.e., elastic (G′) and viscus (G″) moduli. The frequency was in the range of 0.01 to 10 Hz. The measurements were carried out through a controlled stress rotational rheometer (Mars III, HAAKE Rheometer, Waltham, MA, USA), using a parallel plate geometry. In order to identify the linear viscoelastic response range of the materials, preliminary strain sweep tests were performed on the samples, at an oscillation frequency of 1 Hz. The tests were repeated at least three times on each sample. The viscoelastic properties of the formulations were assessed by small-amplitude oscillatory shear experiments using a plate–plate geometry with a 35 cm diameter.

#### 2.4.2. Continuous Shear Rheometry

Flow analysis of the preparations was carried out at 25 °C using a rheometer with parallel steel plate geometry. Samples were carefully applied to the lower plate, making sure that the shear of the formulations was minimized, and at least 1 min of rest was allowed for equilibration before the analysis. In flow mode, the downward and upward curves were evaluated over shear rates. To calculate the thixotropic area, a flow curve was generated to evaluate the dependence of the viscosity upon the shear rate. The shear rate was first increased from 0 to 100 s^−1^ and then kept constant at a maximal speed of 100 s^−1^ before being eventually reduced from 100 to 0 s^−1^, each time within 180 s.

The correlations between the viscometric functions (steady shear flow properties) and linear viscoelastic functions (dynamic viscoelastic properties) were examined by introducing a modified form of the Cox–Merz rule through a comparison of the steady shear viscosity with the complex viscosity [18].

#### 2.4.3. Creep Recovery Analysis

In the creep recovery test, the sample was exposed to the stress at 10 Pa for 180 s, and in the recovery phase, the applied stress was suddenly removed and the sample was analyzed for recoverable shear during 180 s. Each measurement was replicated three times.

### 2.5. Free Radical Scavenging Assay

To evaluate the radical scavenging ability of the formulations, 2,2-diphenyl-1-picrylhydrazyl (DPPH) was used as it has been widely employed to investigate the antioxidant activity of different systems, e.g., dermatological formulations and biomedical scaffolds [19,20]. In the radical form, the DPPH molecule has an absorbance at 517 nm, which disappears after the acceptance of an electron or hydrogen radical from an antioxidant agent to become a stable diamagnetic molecule [21]. For this purpose, 3 mL of the formulation solution in EtOH with different concentrations was added to 1 mL of an ethanolic solution of DPPH (25.0 μg/mL) in a vial. The resultant mixture was shaken thoroughly and allowed to stand at room temperature in a dark place for 1 h. Subsequently, the absorbance of the samples was measured using a UV-visible spectrophotometer (Cary 100 scan, Varian, Palo Alto, CA, USA) to measure the optical density (OD) at 517 nm. Ethanol and DPPH solution were used as the blank and negative control, respectively. The following equation was used to calculate the percentage of scavenging:

(1) $$Scavenging\;ability\left(\%\right)=\frac{A_{control}-A_{sample}}{A_{control}}\times 100$$

where *A_sample_* is the absorbance in the presence of samples and standard and *A_control_* is the absorbance of the control.

### 2.6. Cell Culture

In order to test the biological response to our formulations, primary human dermal fibroblasts (HDF, provided by Lonza) were used. The HDF cells were cultured at passage 5–6 with a complete medium, composed of Eagle’s minimal essential medium (EMEM) supplemented with 20% FBS, 100 U/mL penicillin, 100 U/mL streptomycin and 2X non-essential amino-acids. The HDF cells were maintained in 100 mm-diameter cell culture dishes in a humidified and controlled atmosphere at 37 °C and 5% CO_2_. The medium was changed every 3–4 days.

### 2.7. Cell Viability and Morphology

The viability of the formulation samples (350 µL/Transwell insert) was also evaluated using Transwell inserts (MW6 8 µm polycarbonate). A HDF cell monolayer were seeded confluently in three different 100 mm-diameter culture dishes, one for the control cells, one for testing the HA-based formulations and one for testing the control formulations. Each of the formulations (0.35 mg/4.67 cm^2^) was positioned onto different Transwell inserts. Each insert, including the empty one for the control cells, was then placed on one side of the respective culture dishes containing confluent monolayer cell cultures. Then, cell culture medium (10 mL) was added to the monolayer of confluent cells, seeded into the 100 mm-diameter dishes, containing the inserts inside the respective formulations.

After 24 h of incubation with the test formulations, the cells were washed with 1X Dulbecco’s phosphate buffer solution (PBS) to remove the non-adherent dead cells. The adherent live cells were stained with crystal violet dye, which binds to proteins and DNA. Cells that undergo cell death lose their adherence and are subsequently lost from the population of cells, reducing the amount of crystal violet staining in a culture. The staining is directly proportional to the cell viability. The HDF cells were fixed with 10% buffered formalin for 30 min and washed with ddH_2_O. Crystal violet was added at 0.5% in ethanol, for 15 min. After three washes in ddH_2_O, the dye was eluted with 0.1% SDS (2 mL) and the OD at 545 nm was measured. The percentage (%) crystal violet OD, directly proportional to the cell viability, was determined by comparing the average OD545 values of the stimulated cells with the OD545 values of the control cells [22,23].

Cell morphology was investigated with a confocal laser scanning fluorescence microscope (CLSM). For CLSM analysis, the HDF cells were seeded at a 5000 cells/cm^2^ density. After 24 h, the HDF cells were fixed with 4% paraformaldehyde for 20 min at RT, rinsed twice with PBS buffer, and incubated with Triton 0.1% for permeabilization and afterward with PBS-BSA 0.5% to block non-specific binding. Actin microfilaments were stained with phalloidin–AttoRho6G (Sigma-Aldrich) diluted in PBS-BSA 0.5% by incubating the cells for 30 min at RT. Nuclei were stained with DAPI (blue). The cells were then rinsed three times with PBS and observed with a CLSM (Leica) using a 20X objective.

### 2.8. In Vitro Scratch Assay

The wound healing potential of the formulations was assessed by the wound healing assay [24]. The wound healing assay was performed by scraping the HDF cell monolayer in a straight line to create a “scratch” with a p200 pipet tip. The debris was removed by washing the cells once with 1 mL of the growth medium, and then it was replaced with 2 mL of culture medium that is specific for this in vitro assay. This assay medium was composed of a lower percentage of FBS (2%) than that used in the growth medium, to minimize cell proliferation but be sufficient to prevent apoptosis and/or cell detachment. After the scraping, 0.35 mg of each formulation was positioned into the Transwell inserts (MW6 8 µm polycarbonate) and incubated in each well at 37 °C for 24 and 48 h, and at each time point, a scratched cell monolayer without formulation was used as a control. To study the HDF cell migration, crucial to obtain the wound closure, images were acquired at time zero, immediately after the scratch, and after 24 and 48 h for each sample in triplicate, using bright-field microscopy. At different times, the wound area was calculated using the ImageJ public domain software. The percentage of wound area reduction or wound closure, an expression of the cell migration rate, can be expressed as:

(2) $$Wound\;Closure\;\%=\frac{\left(A_{t=0}-A_{t}\right)}{A_{t=0}}\;\times 100$$

where *A_t_*_=0_ is the area of the wound measured immediately after scratching and *A_t_* is the area of the wound measured after the scratch is performed. The closure percentage increases as cells migrate into the scratch over time.

### 2.9. Statistical Analysis

Statistical measurements were performed using the Prism (version 6.01) software. The results are presented as means ± standard error, and one-way ANOVA was performed, with significance reported when *p* < ±0.05.

## 3. Results and Discussion

### 3.1. Physical Assessments and pH Determination

The color of the formulation was pastel green, Pantone 358 C (R: 173, G: 220, B: 145; C: 34, M: 0, Y: 42, K: 0); however, the smell slightly changed due to the green tea. Thus, it was preferred to add two drops of vanilla essence to mask the bitter smell of the green tea, and it did not alter the properties of the formulation and gave an intense and delicate note. Green tea contains numerous polyphenols such as catechins, which possess antioxidant properties [25]. A number of studies have demonstrated that the catechins prevent the collagenase and tyrosinase activity, resulting in the improvement of skin health [26,27]. In addition, it has been shown that green tea is effective for skin hydration in the elderly [28]. Here, green tea has dual functions including antioxidant and hydrating effects [29]. With an increase in age, skin dryness enhances several skin diseases, e.g., atopic dermatitis, irritation, and allergic contact dermatitis as well as ichthyosis and psoriasis [30]. Hence, moisturizing formulations may be helpful to hinder or overcome this issue. In a study conducted by Tjandra et al. [28], it was reported that green tea-embedded moisturizing topical formulations have considerably more effect on skin hydration than those creams containing vitamin E.

**Figure 1:**
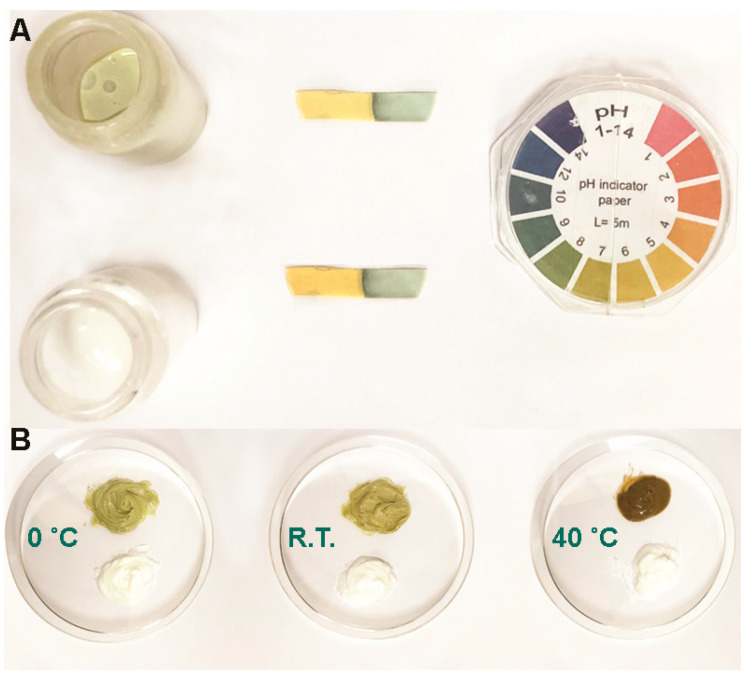
(**A**) Stability test for the herbal and base creams at different temperatures. (**B**) pH determination for the herbal and base creams diluted in double-distilled water.

The stability test consists of monitoring the changes of the sealed formulations subjected to three different temperatures for up to 30 days to evaluate the physical appearance, which is controlled by phase separation, chromatic change, and fragrance. All formulations remained unchanged and maintained stable conditions in the different temperatures except the HBN formulations, which underwent oxidation that was exhibited from the physical point of view by a change in the color and viscosity (Figure 1A). In addition, the pH of both solutions was approximately 7 to 8 as shown in Figure 1B. Therefore, no pH change occurred after mixing the new materials into the base formulation.

### 3.2. Rheological Properties

**Figure 2:**
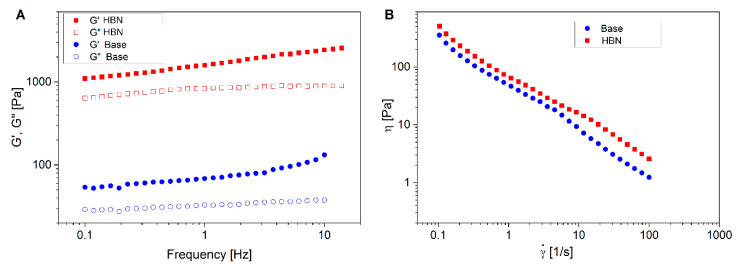
(**A**) Mechanical spectra of the control system (base) and the formulation at 25 °C. (**B**) Shear rate-dependent viscosity changes in the control and the formulation at 25 °C. Error bars were always lower than 10%. The error bars are omitted for clarity purposes.

Oscillatory rheological features are capable of simulating the materials’ performance in response to physiological oscillatory movements, which can affect their efficacy [31]. Figure 2A shows the mechanical spectra of the formulations. The elastic (G′) and viscous (G″) moduli at 1 Hz are presented in Table 1. As can be seen, the HBN formulation possesses higher G′ (1592 versus 62 Pa) and G″ (856 versus 32 Pa) than the control one. The flow curve, which represents viscosity as a function of shear rate, is shown in Figure 2B. Both formulations behave like gel materials as G′ > G″. The experimental formulation has slightly more viscosity than the control formulation. The enhanced mechanical properties and viscosity of the HBN formulation compared to the base one is due to the presence of HA; it has been reported that [32,33] HA improves the viscoelastic properties of systems, e.g., injectable hydrogels [24,34].

For both formulations, the viscosity underwent a reduction upon the shearing over the whole range of shear rates tested (Figure 2B). Upon increasing the shear rate, hydrodynamic forces cause aggregates to become deformed and eventually disrupted and molecule alignment, which results in a decrease in the viscosity [35]. These results indicate that both formulations are non-Newtonian fluids and show shear-thinning behavior, as the viscosity depends on the rate of shear. The observed viscosity decrease may be interpreted by the changes in the microstructure and alignment of polymer chain segments. The reduction of viscosity at a high shear rate is a preferred property of HBN formulations when applied to the human skin so that they can be easily, smoothly spread onto the skin [33].

The Cox–Merz rule (Equation (1)) is a relationship predicting that the complex viscosity |η*(ω)| and steady shear viscosity η(γ) are equivalent when the angular frequency ω is equal to the steady shear rate.

(3) $$\left|\eta^{*}\right|\;\left(\omega \right)=\eta \gamma {|}_{\omega =\gamma }$$

**Figure 3:**
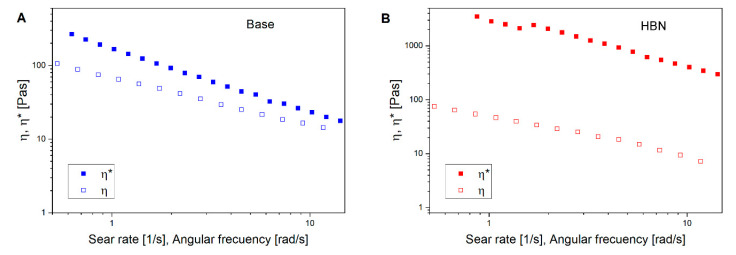
Comparison of shear viscosity with complex viscosity for (**A**) the control and (**B**) the formulation.

Figure 3 shows the comparison between the complex viscosity (against angular frequency) and apparent viscosity (against shear rate). As can be seen, both complex viscosity and shear steady viscosity show the same trend, that is, a reduction with an increase in the angular frequency and shear rate. In addition, the η* is greater than the η over a whole range of shear rates and angular frequencies. The difference between η* and η is more significant for the HBN formulation than the base one. This indicates that the Cox–Merz rule is not appropriate for defining the relationship between the steady shear flow and dynamics. This is attributed to a structural decay owing to the extent of the strain magnitudes applied to the materials, since both formulations undergo severe structural breakdown beyond a certain critical strain magnitude in steady shear rheometry [18].

**Figure 4:**
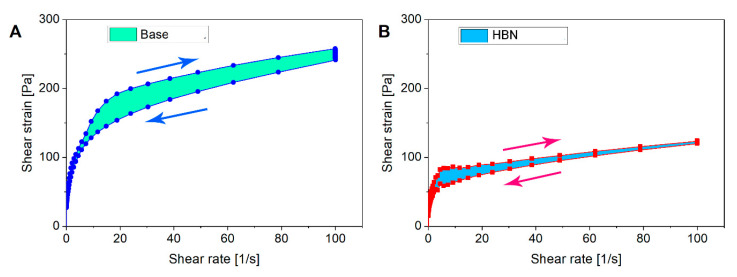
Flow curves of the control and the formulation at 25 °C to calculate the hysteresis area. Error bars were always lower than 10%. The error bars are omitted for clarity purposes. The experiments were repeated three times. (**A**) Base; (**B**) HBN.

A range of performance factors, e.g., easy administration and the time-dependent recovery of the formulation after topical application, can be evaluated by continuous shear (flow) rheometry [36]. Flow curves (with ascending and descending curves) facilitate the definition and analysis of the thixotropy and hysteresis area of the formulations. The thixotropy can be assessed by calculating the surface area of hysteresis on the rheogram (shear stress versus shear rate) as presented in Figure 4. The rheological parameters and the calculated thixotropy are shown in Table 1. A greater hysteresis (surface) area indicates a more time-dependent behavior of the control formulation as it has a higher area (2557 Pa/s) than the HBN formulation (936 Pa/s). The lower area of the thixotropy hysteresis loop indicates better rheological stability [35]. Consequently, the less thixotropic behavior of the HA-based formulation corresponds to its ability to rebuild the damaged structure faster after the removal of shear forces. The viscosity of thixotropic compounds does not follow the same path of structure breakdown and recovery [37,38]. Thixotropy manifests in the ability of the platform to return to its initial structure after the elimination of applied tension. Both platforms display thixotropic behavior in which they lose structure during shear and rebuild it while standing. A topical formulation possessing a thixotropic property is highly desired, since it would become more fluid during applications—so that it can be easily applied to the face and body skin through structure breakdown in spreading, hence resulting in easier spreading—but recover to its initial viscosity after rubbing to prevent formulation leakage [39,40].

**Figure 5:**
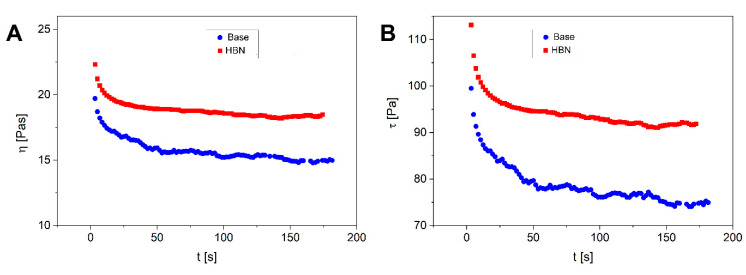
Viscosity (**A**) and shear stress (**B**) of the HBN and base formulation over time at a constant shear rate of 5 Pa. Error bars were always lower than 10%. The error bars are omitted for clarity purposes.

To analyze the time dependency of the viscosity, the flow curve was evaluated at a constant imposed shear rate (Figure 5A,B). Both formulations exhibited a time dependency of viscosity, as typical behavior of thixotropic fluids. The viscosity of thixotropic fluids declines over time under an imposed constant shear rate. If allowed to rest, a thixotropic formulation reaches its initial higher viscosity value. Regarding the dermatological applications and, in particular, topical use, a formulation should provide ease of application to consumers and patients by fast flowability and deformation [32,38]. Therefore, this feature is desirable for dermatological formulations as they can easily be distributed with the low stress applied by consumers upon rubbing on the skin [37].

**Figure 6:**
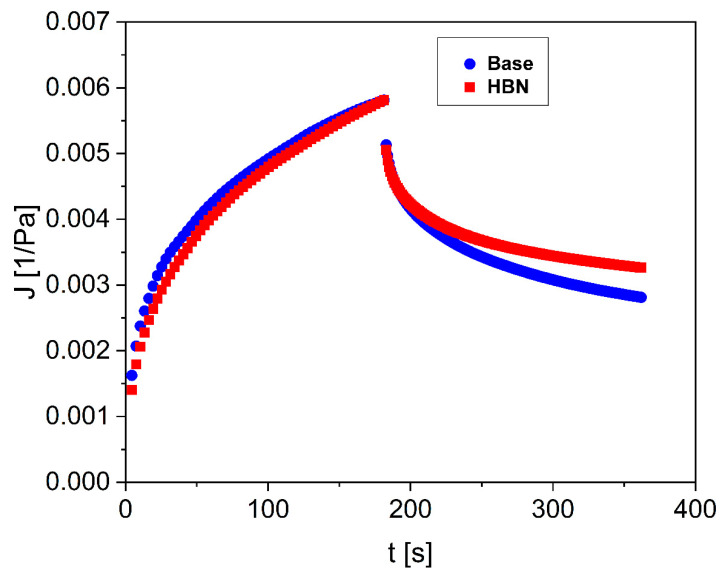
Creep-recovery curves of the control and the formulation. The change in compliance versus time is illustrated. Error bars were always lower than 10%. The error bars are omitted for clarity purposes. The experiments were repeated three times.

The creep-recovery test provides information about the deformation and recovery of the formulations when subjected to a predetermined tension for 180 s. After the removal of the imposed stress, recovery measurements were carried out for up to 180 s. The change in compliance and viscosity with time is demonstrated in Figure 6. Upon the cessation of the shear that caused the breakdown, the HBN formulation reforms its internal network (structure build-up), and the viscosity recovers. As it can be seen, there is no difference between the recovery and j_max_ of both systems (Table 1). It stands to reason that the recovery state depends on the stabilization of internal network structures that can be broken down by shearing and require time to rebuild [41]. In addition, the thixotropy area and recovery test demonstrated that the apparent viscosity decreases under shear stress, followed by a gradual recovery when the stress is eliminated.

### 3.3. Antioxidant Activity

**Figure 7:**
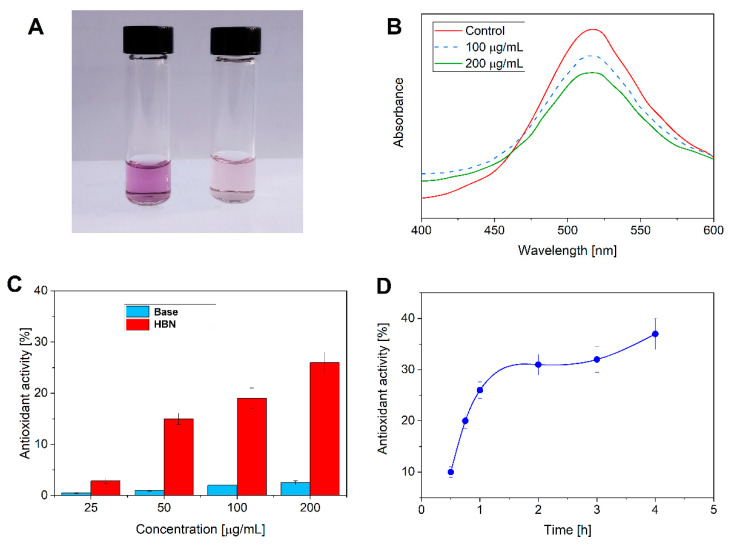
(**A**) Photograph of samples with Base (left) and HBN formulation (right). (**B**) UV-visible spectra of the control and samples containing 100 and 200 µg/mL. (**C**) Effect of concentration on the antioxidant activity. (**D**) Effect of time on the antioxidant activity of solutions containing 200 µg/mL.

The antioxidant ability of the formulations to scavenge reactive radicals was investigated using the DPPH assay (Figure 7). DPPH is a stable nitrogen-based free radical, which has a violet color that changes to yellow after reduction by the process of hydrogen- or electron-transfer [42,43].

Figure 7A shows the optical images of the solution without and with the HBN formulations, and the qualitative UV-visible spectra of the control and samples containing 100 and 200 µg/mL are shown in Figure 7B. As can be seen, the solutions containing the HBN formulations (100 and 200 µg/mL) have lower absorbance than the control. In addition, upon increasing the concentration of the formulation (from 100 to 200 µg/mL), the absorbance is reduced. Increasing the concentration of the formulation leads to an enhancement of the antioxidant properties whereas the control did not show significant antioxidant activity (Figure 7C). The results indicate that the HBN formulation possesses antioxidant activity while the control formulation does not have this capability. In addition, the time has effects on the antioxidant ability of the formulation, in which the antioxidant activity is increased over time (Figure 7D).

The production of reactive radicals is significantly increased during cell metabolism and dermal tissue regeneration mechanisms [7]. High concentrations of these reactive species are present in wound sites, inducing harmful effects on cells and tissues and even promoting oxidative stress, which generates lipid peroxidation, damage to deoxyribonucleic acid (DNA), and enzyme inactivation, including that of free radical scavenger enzymes. In this frame, the antioxidants may represent potential therapeutic tools to enhance and accelerate the wound healing process. Antioxidants are postulated to help control wound oxidative stress and thereby accelerate wound healing. They are important mediators in regulating the damage that is potentially incurred by biological molecules such as DNA, protein, lipids, and body tissue in the presence of reactive species [44,45].

The antioxidant activity of the formulation comes from the raspberry juice and vitamin E. Raspberry juice has also been reported as being an effective antioxidant, which is due to the presence of phenolic and flavonoid compounds [46]. Flavonoids, which are polyphenolic substances, protect the organism from oxidative damage produced from UV rays, chemical reagents, and environmental contamination [47]. Vitamin E, the most abundant antioxidant in the skin, is located in the cellular membranes and acts within cells to provide antioxidant protection. The antioxidant potential shows that the formulation has a higher percentage free radical scavenging potential, which positions it as an excellent candidate for the wound healing process [48].

### 3.4. Biocompatibility and Wound Healing Potential 

**Figure 8:**
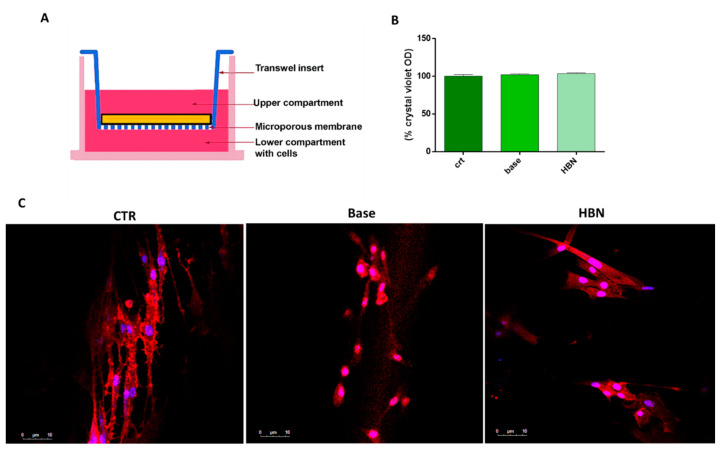
Transwell inserts (**A**) and the % crystal violet OD, directly proportional to cell viability of control cells and cells exposed to HBN formulation in comparison with those exposed to the base formulation (**B**). (**C**) Human derived fibroblast (HDF) cell morphology for the control cells, base formulation and hyaluronic acid (HA)-based formulation after 24 h. In red are Actin filaments stained by AttoRho6G–phalloidin, and in blue are DAPI-stained nuclei cells. Bars represent 10 µm.

In order to investigate the biocompatibility and the safety of the topical formulation based on HA and natural compounds, the crystal violet assay was performed on HDF cells. The assay was conducted by using a Transwell insert (Figure 8A). Figure 8B shows the % crystal violet OD (absorbance at 545 nm). As can be seen, the results revealed good biocompatibility of the both the base formulation and HBN formulation, with values of around 100% after 24 h of exposure compared with the untreated control cells. The formulations, indeed, were not harmful to cells and, moreover, were able to positively affect their viability. The biocompatibility of the topical formulation was also confirmed from a morphological point of view. Indeed, as shown in Figure 8C, the HDF cells in contact with the HA-based formulation show a shape typical of healthy in vitro fibroblast cells. The cytoplasmatic morphology (Ph Red) of the cells appears to be similar after 24 h of contact for both of the formulations (base and HA natural-based formulation), compared with the control cells. The receptor for hyaluronan-mediated motility is extensively expressed in fibroblasts, and it has been proven that the activation of this receptor stimulates fibroblast viability in vitro [46], so the HA-based formulation can naturally keep cells safe and improve their viability.

The wound-healing ability of the skin is an imperative physiological process for maintaining integrity after trauma, either by accident or by an intentional procedure, and the process induces cell migration, proliferation, and inflammatory responses. Aberrations of wound healing, such as excessive wound healing (hypertrophic scars sand keloids) or chronic wounds (ulcers), impair normal physical function [49,50]. After wound injury, a large number of cytokines are released, which promote the migration, proliferation, and survival of various cell types, such as fibroblasts and keratinocytes, at the wound site. Indeed, the re-epithelialization process after the injury to the epidermis in a skin wound is initiated by keratinocyte and fibroblast migration, followed by the proliferation of these cells and their subsequent redifferentiation [49]. To evaluate the potential wound healing activity of the HA-based natural formulations, an in vitro scratch assay on HDF cells was performed. The presence of HDF cells in the “wounded” or scratched area due the migration of cells and also the proliferation of the migrated cells was verified after 24 and 48 h of treatment with the formulations [50].

**Figure 9:**
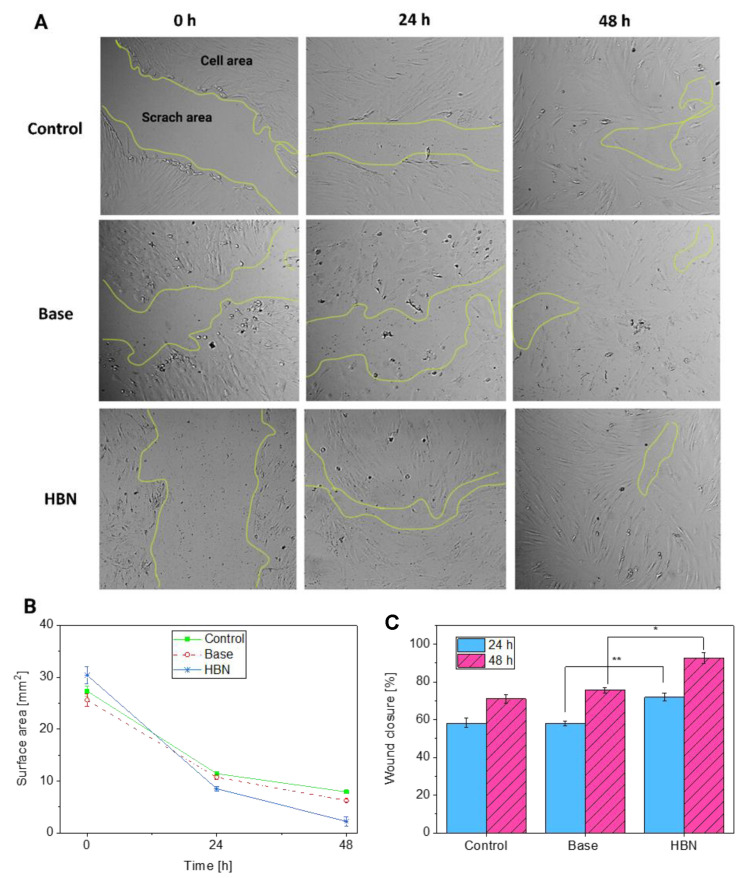
(**A**) Representative bright-field images show HDF cell migration after the scratch at time 0 and after 24 and 48 h of incubation with the HDF control cells and formulations. (**B**) The wound area expressed as the remaining area uncovered by the cells. The scratch area at time point 0 hours, and after 24 and 48 h of incubation with the formulations. (**C**) Wound closure expressed as the percentage of the closure of the scratched gap after 24 and 48 h of incubation with the formulations. All experiments were repeated 3 times in triplicate. The results are the means of three measurements. *, ** *p*-Value < 0.05 compared to the control. Clinical skin test was used to evaluate the contact allergy of the patches containing the herbal and base creams (Figure S1).

Figure 9A shows the representative bright-field images of HDF cells, immediately after reproducing the scratch area (time 0) and after 24 and 48 h of incubation with the base and HA-based formulations, compared with the control without formulation [51]. The wound surface area (Figure 9B) decreases from 24 to 48 h for both the formulation and control without formulation. The cells with the control base formulation and control cells without formulation exhibited a similar trend during the time, showing a decrease in wound surface area from about 30 to 10 mm^2^ after 24 h and to around 7 mm^2^ after 48 h. The HA-based formulation induced a bigger decrease in the wound surface area, which decreased to below 8 mm^2^ after 24 h and to around 2 mm^2^ after 48 h of contact with the cells. Accordingly, the wound closure percentage (Figure 9C) was much higher for the HA-based formulation than the control formulation and HDF control at both 24 and 48 h of time. In particular, after 24 h, the wound closure percentage for both the HDF control and the control formulation was about 60%, whereas for the prepared HA-based formulation, it was about 70%; at 48 h, the wound closure percentage was about 70% for the HDF control and 75% for the control base formulation, while for the HA-based formulation, it was around 92%. These results suggest that the specific components of the HA-based formulation are effective. HA and natural compounds are able to positively improve the migration capability of the HDF cells, thus making the process of closing the wound scratch faster than that observed with the control formulation and HDF control cells. Here, HA is an essential component of the formulation, and thanks to its viscoelastic properties, it is suitable for topical use, so it can be applied either as a cream or as a dressing, facilitating the healing of acute and chronic wounds. Indeed, HA was used for its wound healing potential, HA facilitated re-epithelialization, and it is able to accelerate the physiological process. It was shown that HA with low molecular weight, approximately between 20 and 300 kDa, can penetrate the stratum corneum whereas high molecular weight HA (1000–1400 kDa) exhibited impermeability [52]. Moreover, HA led to an enhancement of cell migration, allowing the formation of soft tissue with good elasticity, and increased microvascular density. In particular, the healing process may be explained by the HA action during the re-epithelialization phase. HA activates the proliferation and migration of keratinocytes and promotes dermal collagen remodeling during morphogenesis [53].

## 4. Conclusions

In this study, HA-based formulations were enriched with ingredients exhibiting antioxidant activity including vitamin E and natural compounds, such as raspberry and green tea, to create a formulation to promote the healing process in topical applications. The HA-based formulation has a higher viscosity and elastic modulus than the control formulation. The HA-based formulation also showed good antioxidant activity in a concentration- and time-dependent manner, being able to hinder reactive radicals produced during cell metabolism and dermal tissue aging mechanisms. The results of cell viability and morphological analysis demonstrated the biocompatibility of the formulations. Moreover, to evaluate the potential wound healing activity of the HA-based natural formulations, an in vitro scratch assay on HDF cells was carried out. The presence of HDF cells in the scratched area due to the migration of cells and also the proliferation of the migrated cells showed the potential ability of the HA-based formulation, in comparison with base formulation and observations with control cells, to further promote the wound healing process. In this work, the presence of HA had dual functions including enhancing the viscoelastic properties so as to allow the topical use of the formulation and improving the wound healing ability with the antioxidant action of vitamin E and natural compounds.

## Figures and Tables

**Table 1:** Rheological properties of the control and the experimental formulation.

Sample	Maximum Compliance (j_max_)	Viscosity ^a^ (Pa.s)	G′ ^b^ (Pa)	G″ ^b^ (Pa)	Thixotropic Curve Area (Pa/s)
Base formulation	0.0058	1.4	70	32	2557
HBN formulation	0.0058	2.5	1616	819	936

^a^ viscosity at maximum share rate. ^b^ G′ and G″ at 1 Hz.

## Supplementary Materials

The following are available online at https://www.mdpi.com/2073-4360/12/8/1847/s1, Figure S1: Safety and allergic tests for the base and herbal creams.
