# Supplementary material for: Natural Formulations Provide Antioxidant Complement to Hyaluronic Acid-Based Topical Applications Used in Wound Healing
Source: Polymers (Basel). 2020 Aug 18;12(8):1847. doi: 10.3390/polym12081847 (PMC7465439; doi:10.3390/polym12081847)
Supplement: Supplementary file 1 [file polymers-12-01847-s001.pdf]

Supporting information

Natural Formulations Provide Antioxidant Complement to Hyaluronic Acid-Based Topical Applications Used in Wound Healing

Pooyan Makvandi [1,2], Caterina Caccavale [3], Francesca Della Sala [1,3], Stefania Zeppetelli [1], Rosanna Veneziano [3] and Assunta Borzacchiello [1,*]

1 Institute for Polymers, Composites and Biomaterials, National Research Council, IPCB-CNR, 80100 Naples, Italy; pooyan.makvandi@ipcb.cnr.it (P.M.); fr.dellasala@gmail.com (F.D.S.); stefania.zeppetelli@cnr.it (S.Z.)

2 Department of Chemical, Materials and Production Engineering, University of Naples Federico II, 80100 Naples, Italy

3 University of Campania "Luigi Vanvitelli", 81100 Caserta, Italy; caterina.caccavale93@gmail.com (C.C.); rosanna.veneziano@unicampania.it (R.V.)

* Correspondence: bassunta@unina.it

Skin test

Clinical skin test was used to evaluate the contact allergy of the patches containing the herbal and base creams. To this aim, five applicants including three females (18, 25, 64 years old) and two males (28 and 32 years old) applied the cream patches. The results showed no sign of any redness or inflammation to the applicants which indicate that the cream does not show allergy for the consumers (**Fig. S1**). However, it differs from person to person and some consumers may show some allergy.

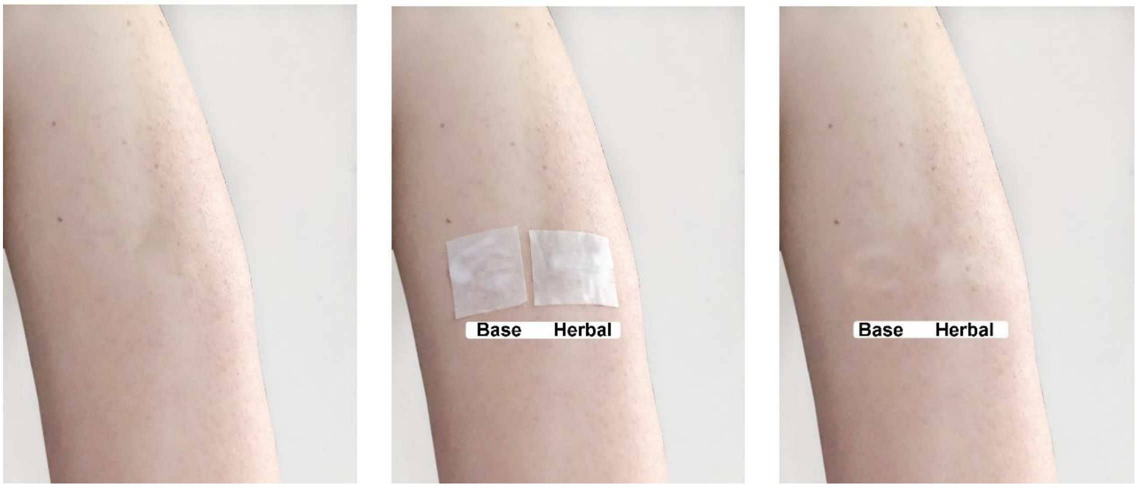

Figure S1. Safety and allergic tests for the base and herbal creams. .
